# Hand-Held Refractometer-Based Measurement and Excess Permittivity Analysis Method for Detection of Diesel Oils Adulterated by Kerosene in Field Conditions

**DOI:** 10.3390/s18051551

**Published:** 2018-05-14

**Authors:** Boniphace Kanyathare, Kai-Erik Peiponen

**Affiliations:** Department of Physics and Mathematics, University of Eastern Finland, P.O. Box 111, FI-80101 Joensuu, Finland

**Keywords:** fuel adulteration, refractive index, excess permittivity, hand-held refractometer, diesel oils, kerosene

## Abstract

Adulteration of fuels is a major problem, especially in developing and third world countries. One such case is the adulteration of diesel oil by kerosene. This problem contributes to air pollution, which leads to other far-reaching adverse effects, such as climate change. The objective of this study was to develop a relatively easy measurement method based on an inexpensive, handheld Abbe refractometer for the detection of adulteration and estimation of the ascending order of the amount of kerosene present in adulterated samples in field conditions. We achieved this by increasing the volume of pure diesel sample in the adulterated diesel oil, and measuring the trend of refractive index change, and next, exploiting the true and ideal permittivities of the binary mixture. The permittivity can be obtained with the aid of the measured refractive index of a liquid. Due to the molecular interactions, the true and ideal permittivities of diesel–kerosene binary liquid mixture have a mismatch which can be used to screen for adulterated diesel oils. The difference between the true and the ideal permittivity is the so-called excess permittivity. We first investigated a training set of diesel oils in laboratory in Finland, using the accurate table model Abbe refractometer and depicting the behavior of the excess permittivity of the mixture of diesel oil and kerosene. Then, we measured same samples in the laboratory using a handheld refractometer. Finally, preliminary field measurements using the handheld device were performed in Tanzania to assess the accuracy and possibility of applying the suggested method in field conditions. We herein show that it is not only possible to detect even relatively low adulteration levels of diesel in kerosene—namely, 5%, 10%, and 15%—but also it is possible to monitor the ascending order of adulteration for different adulterated diesel samples. We propose that the method of increasing the volume of an unknown (suspected) diesel oil sample by adding a known authentic diesel sample and monitoring excess permittivity is useful for the screening of adulterated diesel oil in field measurement conditions.

## 1. Introduction

Liquid fuel adulteration is a serious problem in developing countries but also in some parts of Europe [1,2,3], and it is also one reason for air pollution. The illegal practice of mixing either diesel and kerosene or gasoline and kerosene tends to be the most typical method of fuel adulteration in many parts of developing countries [2,3].

The measurement of liquid fuel purity is of high importance especially for fuel quality inspection for engines and to screen for fuel adulteration. In recent years, optics-based liquid fuel quality research has become even more important due to the development of biodiesels and bioethanol products, as demonstrated in [4,5,6]. Because fuel adulteration involves a change in liquid purity, the study of purity is of high importance for the detection of fuel adulteration, such as diesel adulteration with kerosene. The refractive index of a liquid has been used for a long time in studies of liquid purity in various industrial sectors, such as petroleum, chemical, and pharmaceutical, to mention a few. Moreover, optical devices of different kinds and also different analytical methods have been developed for the inspection of the refractive index and hence, the purity of liquids [7,8,9,10,11,12]. In the case of diesel oil that has undergone adulteration by kerosene, the purity is worse than that of authentic diesel oil and this is reflected by a higher air pollution rate from diesel engines. The refractive index of a liquid is an intrinsic property and it depends not only on the wavelength of probe electromagnetic radiation, such as light, but also on the temperature and pressure of a liquid. Therefore, the refractive index is a useful measure for the identification of the purity of liquids. In the case of diesel oil adulterated by kerosene, the mixing of the two liquids results in a binary liquid mixture which is in thermodynamic equilibrium. Typically, diesel oil and kerosene have different densities and hence, they usually have different refractive index values. However, this is not always the case as will be shown in this study. Furthermore, intermolecular interactions between different hydrocarbons present in diesel oil and kerosene contribute to the dipole moments of electric charges. The probe light, in turn, interacts with the electrons of a binary fuel mixture. As a result, the refractive index of a binary liquid may not fulfill traditional mixing rules for the refractive index and permittivity of a binary liquid mixture. One of the major challenges in the study of diesel adulteration by kerosene is the fact that unlike gasoline and kerosene, diesel and kerosene can have refractive index values that are very close to each other. This complicates the adulteration detection process especially when the volume of kerosene in diesel is below 20%. Furthermore, the existing ideal liquid mixture laws, such as Arago–Biot, Newton, and Lorentz–Lorenz [13], tend to overestimate the volume fraction of kerosene in diesel and cannot be considered reliable, especially for the determination of the exact amount of kerosene in, for example, diesel oils adulterated with kerosene.

In our recent work, we developed a prototype of a hand-held sensor for adulterated diesel oil screening based on refractive index mismatch between a roughened glass probe window and the sample liquid, but this device needs high mechanical stability [14]. Furthermore, we developed another method for the detection of diesel adulteration based on light dispersion theory [15]. However, that method requires a combination of sophisticated measuring equipment, such as a spectrophotometer and an accurate table model Abbe refractometer, as well as Kramers–Kronig analysis of the measured data. We have shown these methods to be useful [15] for the assessment of the wavelength-dependent relative excess permittivity of a binary liquid [16] to screen for problematic adulterated samples. Unfortunately, these instruments are not practical for field conditions and are rather expensive, especially for poor countries where fuel adulteration is a major issue. Therefore, we wish to introduce a method that is not only simple but also reliable for the detection of diesel oil adulteration. This method only requires an inexpensive hand-held refractometer, and it is shown that the concept of excess permittivity—which was recently introduced in adulterated diesel oil screening [15]—can be exploited using a hand-held refractometer together with the novel method introduced in this article. Furthermore, unlike the table model refractometer, spectrophotometer and chemometrics used for data analysis, which require highly-trained personnel, the hand-held device is straightforward to use. In this work, we introduce a novel method that is based on increasing the volume of a pure diesel oil sample into the binary mixture of adulterated diesel oil and measuring the trend of the refractive index change. The analysis is based on the exploitation of the true and ideal permittivities of the binary liquid mixture and hence, deals with excess permittivity. The excess permittivity has been suggested as a rigorous measure for the analysis of properties (in our case, optical) of binary- and multi-liquid mixtures [17]. The protocol of the measurement is rather simple, namely the refractive index of the suspected sample is measured and the measurement is repeated after the original sample’s volume has been increased with authentic diesel oil. The viability and potential of this method were first confirmed by measurements of training sets in the laboratory using both table and hand-held models of Abbe refractometers. Thereafter, the method based on the hand-held refractometer was applied for field measurements in Tanzania.

## 2. Theory

The mixture of diesel oil and kerosene is a binary liquid mixture. According to [13], the refractive index for a binary mixture of a given composition can be successfully approximated using several well-known mixing rules. However, the typical formula in liquid studies, whose validity we have tested in this study, is the Lorentz–Lorenz formula, expressed as follows:

(1) $$\frac{n^{2}-1}{n^{2}+2}=f_{D}\frac{n_{D}^{2}-1}{n_{D}^{2}+2}+\left(1-f_{D}\right)\frac{n_{K}^{2}-1}{n_{K}^{2}+2},$$

where *n*, *n_D_* and *n_K_* are the refractive indices of the mixture, diesel oil, and kerosene respectively, and *f_D_* is the volume fill fraction of diesel oil in the mixture. Using this model, an ideal mixture is defined that behaves according to the chosen rule.

Next, we wrote another expression with the aid of the volume average of ideal relative permittivity of a binary mixture for the case of diesel adulterated by kerosene, as follows [16]:

(2) $$\varepsilon^{ideal}=f_{D}\varepsilon_{D}+\left(1-f_{D}\right)\varepsilon_{K},$$

where *ε^ideal^* is the ideal permittivity of the mixture, *ε_D_* is the permittivity of diesel oil and *ε_K_* is the permittivity of kerosene. We re-expressed this equation to be written in terms of the volumes occupied by diesel and kerosene as proportions of the total volume, therefore Equation (2) became

(3) $$\varepsilon^{ideal}=\frac{V_{D}}{V}\varepsilon_{D}+\frac{V_{K}}{V}\varepsilon_{K},$$

where the total volume is *V = V_D_ + V_K_*. In this initial system, everything is constant, namely, the fill fractions and the intrinsic permittivities. In the measurement protocol, the idea was to mix suspected fake diesel oil with authentic diesel oil. Therefore, we introduced a new variable *V’* to describe the change of the total volume of a sample resulting from increasing the volume of the originally adulterated diesel oil (Equation (3)). Then, we were able to write a modified formula, which incorporates this additional volume, as follows:

(4) $$\varepsilon^{ideal}\left(V^{\prime }\right)=\frac{V_{D}+V^{\prime }}{V+V^{\prime }}\varepsilon_{D}+\frac{V_{K}}{V+V^{\prime }}\varepsilon_{K}.$$

The choice of the magnitude of volume (*V’*) is relatively unrestricted but should be chosen regarding the magnitude of the volume (*V*) of the suspect fuel taken for the measurements. It is obvious that if we continued to increase (*V’*) we would end up getting closer and closer to the ideal mixture case, as the volume of the adulterant is neutralized by the volume of the authentic diesel fuel. The limiting value for this volume addition process is

(5) $$\underset{V\prime \to \infty }{\lim}\varepsilon^{ideal}\left(V^{\prime }\right)=\varepsilon_{D}.$$

In general cases, the permittivity of the liquids can be a complex number, leading to complex permittivity, which depends on the wavelength of light (λ). It is given as

(6) $$\varepsilon \left(\lambda \right)=\varepsilon_{1}\left(\lambda \right)+i\varepsilon_{2}\left(\lambda \right)=n^{2}\left(\lambda \right)-\;k^{2}\left(\lambda \right)+i2n\left(\lambda \right)k\left(\lambda \right),$$

where *n* is the refractive index and *k* is the extinction coefficient. Using the refractive index measurement of the binary mixture, we get the true permittivity (*ε*) of the mixture. In cases of absent or negligible light absorption by an insulator it holds, according to Equation (6), that *ε* = *n*^2^ (note that square of the refractive index also appears in the Lorentz–Lorenz formula). This permittivity is typically different from the *ε^ideal^* and the difference *ε^E^* = *ε − ε^ideal^* is called the excess permittivity, which is considered to be a rigorous measure [16,17] for the intermolecular interactions that have a direct consequence for the magnitude of the refractive index of the binary mixture. The light wave interacts with the electrons of the liquids. Therefore, polarization of the electrons affects both the propagation velocity and attenuation of the light field in the binary mixture. If there is no interaction between the molecules, then *ε^E^* = 0. If *ε^E^* < 0, the polarization of electrons is reduced, whereas if *ε^E^* > 0, there is an increase in the polarization of the charges due to contrasting molecular interactions between molecules of diesel oil and kerosene. The magnitude and sign of the excess permittivity is an indicator of the adulteration of diesel oil. In the case of minute changes in the refractive index between authentic and minimally adulterated diesel oil samples, an adulterated sample might be interpreted as an authentic one. The power of the excess permittivity was demonstrated in the Table 2 in ref. [15], where switching of the sign of ε^E^ was shown to occur for 5% of adulterated samples. Actually, in our studies, we have observed, practically speaking, the same refractive index reading measured with an accurate table model refractometer for rather highly-adulterated (15%) and authentic diesel oil grade (results to be presented elsewhere). In such cases, the strong intermolecular interactions in the binary mixture that reflect the optical properties, such as the refractive index, can lead to the misinterpretation of an authentic “diesel oil”. Thus, the method of volume increase of suspect diesel and the study of excess permittivity improves the reliability in deciding whether a sample is adulterated or not.

In the present measurement process for adulterated diesel oil, the idea is to increase the volume sequentially with portions the size of the initially chosen sample volume. Then, by measuring the refractive index and calculating the true permittivity (*ε*) for this increase in volume of the fuel sample and plotting the true permittivity as a function of increase of volume, it should be possible to monitor the match or departure of the experimental (measured) data from ideal mixture, and hence, screen for the presence of a fake diesel oil. In cases involving an authentic diesel oil, the same permittivity value will be obtained after each volume increase, because in Equation (4), it holds that *V_K_* = 0, and *V* = *V_D_*_._ In contrast, for adulterated diesel oil, we expect to get different values for the true permittivity and then, when the volume is large enough, we should arrive closer and closer to the permittivity of authentic diesel oil.

## 3. Materials and Methods

### 3.1. Materials

Firstly, we studied diesel oils that were processed for utilization in varying European climatic conditions, namely summer and winter. The initial round of refractive index measurements for this study were performed in laboratory conditions in Finland using the table model Abbe refractometer. We purchased from diesel oil samples A and B from a gasoline station. The origin of the crude oil of these diesel oils was Russia. Sample A represents summer diesel oil while sample B (−20 °C), represents winter diesel oil with the lowest temperature of engine operation in parenthesis. The kerosene, sample C, utilized for laboratory studies was a low odor commercial product (Alfa Aesar, Haverhill, MA, USA). Samples A–C were exploited for the preparation of training sets, and adulterations containing kerosene in the proportions of 5%, 10%, and 15% were studied. The typical adulteration level is 20% but we wanted to study lower percentages, which still would be of interest for illegal profit making but are more difficult to screen for than 20% adulteration. The refractive index for each of the fuel samples was measured at room temperature with the aid of the table model Abbe refractometer (Atago RX5000, Atago co. Ltd, Tokyo, Japan) with an operating wavelength of 589 nm, and a relatively high accuracy of ±0.00004 refractive index units, and a hand-held refractometer (Atago H-50, Atago co. Ltd, Tokyo, Japan) which has traditionally been used for glucose concentration measurements.

Secondly, the adulterated samples for outdoor field condition measurements were obtained by blending diesel and kerosene, and the refractive index measurements were performed with a hand-held refractometer. The diesel oil and kerosene samples for the second round of measurements in field conditions in Tanzania were provided by the Energy and Water Utilities Regulatory Authority (EWURA, Dar es Salaam, Tanzania), which is the official government agency for the regulation and control of fuel products in Tanzania. Variation in temperature usually affects refractive index readings due to the thermo-optical constant of the samples. Fortunately, the hand-held refractometer is accompanied by a temperature correction table, which enables the user to take into account any temperature variations in order to provide good estimates of the measured refractive index. 

### 3.2. Methods

In this part, we describe the elaborate step-by-step procedure for the method of diesel adulteration detection using a hand-held refractometer. We start with a diesel oil sample and we want to test whether it is adulterated or not. For this to work we must have authentic diesel oils (tested and approved by fuel regulatory agencies like EWURA) and an Abbe refractometer. This method is based on mixing authentic diesel oil of a measured volume with the suspected sample and measuring the refractive index of the mixture. In the beginning, the volume of the sample is measured, and thereafter, the volume is increased by known amounts of the authentic diesel oil and the refractive index is measured for all added volumes. The process is as follows:

- First measure the refractive indices for both authentic diesel oil and the suspected sample. If the values are exactly same, then the sample is likely to be authentic; however, the measurements should continue as described below.
- After increasing the volume of authentic diesel oil into the sample, the refractive index reading may change. This indicates that the diesel oil sample has been adulterated.
- After observing the change in the refractive index reading, one can go on further increasing the volume of the binary mixture in order to estimate the level of adulteration to confirm whether the sample is highly adulterated or slightly adulterated.
- Because there is a possibility of molecular interactions in the mixtures of diesel oils and kerosene, we utilize the concept of excess permittivity to characterize diesel oils.
- When more and more diesel oil is introduced into a sample, *V’* is used as a variable. The refractive index of the initial sample is measured and corresponds to *n(V’ = 0)*. Then, using different values for *V’* the possibly adulterated samples are measured to determine whether they have the same or different readings of *n(V’)*. Next, *n*^2^*(V’)*, which is the true permittivity, is calculated and this value is plotted together with the value that was obtained from the ideal permittivity law.

For more clarity, we started with the assumption that we had a relatively highly adulterated sample. As an example, let us start with an initial condition that is diesel oil adulterated by 15% of kerosene. For this sample, in the beginning, *V’* = 0, because no volume of authentic diesel has been added yet. Hence, *V_D_ =* 0.85 V, where *V = V_D_ + V_K_*. In the dilution process, we next assume that after adding a certain volume of authentic diesel (*V’*) into the initial sample (diesel sample adulterated by 15% kerosene), the total volume of pure diesel in the mixture increases, while the volume of kerosene remains the same; thus, we assume that this corresponds to 10% adulteration. This means that, in Equation (4), the volume fraction of the diesel oil becomes *f_D_* = (0.85 V + V’)/(V + V’) = 0.9. Using this equation, we solve for *V’* and get *V’ =* 0.5 V. Hence, the total volume of the 10% adulterated sample at this stage is 1.5 V. Furthermore, this 10% sample is next diluted to 5% which gives *V’ =* 2.0 V. Using the measured refractive index data of authentic diesel oil and kerosene and by calculating *ε^ideal^* we can freely choose *V* ’ and plot the resulting ideal permittivity of the binary mixture as a function of *V’*. For the case of experimental data, we get three discrete permittivity points at *V’* = 0, 0.5 and 2.0 in a graphical presentation. The ideal permittivity as a continuous function of *V’* can be calculated using Equation (2) and the refractive index values can be measured from authentic diesel oil and kerosene. Using such data, the excess permittivity can be assessed.

## 4. Results and Discussion

### 4.1. Laboratory Training Set Measurements

In Table 1, the refractive index values from the laboratory measurements are shown. These values were measured at room temperature using the accurate table model Abbe refractometer. Based on the values given in Table 1, is can be observed that the summer diesel oil (sample A) had a higher refractive index compared to the other samples. Usually, when the refractive index value of diesel oil is comparatively higher than that of kerosene, it is possible to detect adulteration more easily than in cases where the refractive indices of two constituent liquids are very close to each other. Such a case holds for samples B and C, namely the refractive indices of sample B is very close to that of sample C. This further complicates the adulteration detection process because we would expect only a minute change in the refractive index and a value between the two authentic samples in the frame of classical binary mixture laws.

Furthermore, in Table 1, the refractive indices of adulterated diesel oils are also shown. It is evident from Table 1 that the refractive indices of sample A adulterated by 5%, 10% and 15% were very close to that of pure diesel sample A. Moreover, the refractive index readings for these adulterated samples were between authentic sample A and kerosene sample C, and apparently follow the assumptions of the conventional binary mixing model. For the case of sample B adulterated by 5%, 10%, and 15%, the refractive index readings were higher than the refractive index of authentic diesel oil. In this case, the assumptions of the conventional binary mixing model were clearly violated, because all the adulterated samples had a higher refractive index than the authentic diesel oil and kerosene. Actually, an excess refractive index is a measure of adulteration, but we prefer a more rigorous measure that is based on the exploitation of the permittivity instead of the refractive index. Regarding the fact that the refractive indices of adulterated sample B diesel oil were very close to that of kerosene sample C, and only differed in the third decimal place, makes it a problematic case for screening for adulteration.

Next, in Table 2, are the estimated fill fractions of authentic diesel oils present in the mixture. These were calculated using the Lorentz–Lorenz model from Equation (1) and the measured refractive index data from Table 1. It is obvious from Table 2 that, for sample A, the volume fraction was well predicted for the 5% case. Unfortunately, the method gave the same value for 10% adulteration as for 5% adulteration. This is obvious because the refractive index values for 5% and 10% differed only in the fifth decimal place. Furthermore, the value that we would expect to be for 10% was given by Equation (1) for the 15% adulteration sample. Actually, the failure of the Lorentz–Lorenz model to correctly estimate the fill fraction is not a shortcoming in this case, but rather, it is a strength because it tells us that the samples are adulterated, and by utilizing the readings of the table model Abbe refractometer and the Lorentz–Lorenz model, we can approximate, at least, the level of adulteration within 5–10%. The discrepancy of the calculated fill fraction with the true fill fraction is higher for higher kerosene volume fill fractions, such as 15%. The discrepancy of the diesel oil fill fraction is due to the excess permittivity (or excess refractive index) which the Lorentz–Lorenz law does not consider.

For the case of sample B, the calculated volume fractions were not realistic, and this was expected because the refractive indices for the 5%, 10%, and 15% samples were above the refractive index value of the authentic sample. Actually, the intermolecular interactions have to be relatively strong to increase the refractive index of the adulterated samples—higher than that of B and C. From this observation, it is obvious that the behavior of different fuel samples is different, and even with the accurate table model Abbe refractometer it can be difficult to conclude the exact levels of adulteration.

Next, we show permittivity data for the training sets measured in the laboratory, namely, samples A, and B adulterated by kerosene. We show both the ideal permittivity and the measured permittivity, starting with the 15% adulterated sample, which had, in this case, an initial value of *V’* = 0, and thereafter, the sample volume increased by 0.5 V and 2 V. In the measurements, the order of magnitude of the sample volume is in ml. Since the data were obtained at 589 nm, the absorption of light of the samples was low, and hence, the imaginary part of the refractive index had only a negligible role in the calculation of the real part of the permittivity. Thus, it was possible for the extinction coefficient to be neglected in the calculation of the real part of the permittivity from Equation (6) [15]. However, due to coloring agents, diesel oil can absorb light at 589 nm. Thus, for better accuracy, one may take into account the extinction coefficient. However, this was not the case with our present samples.

**Figure 1:**
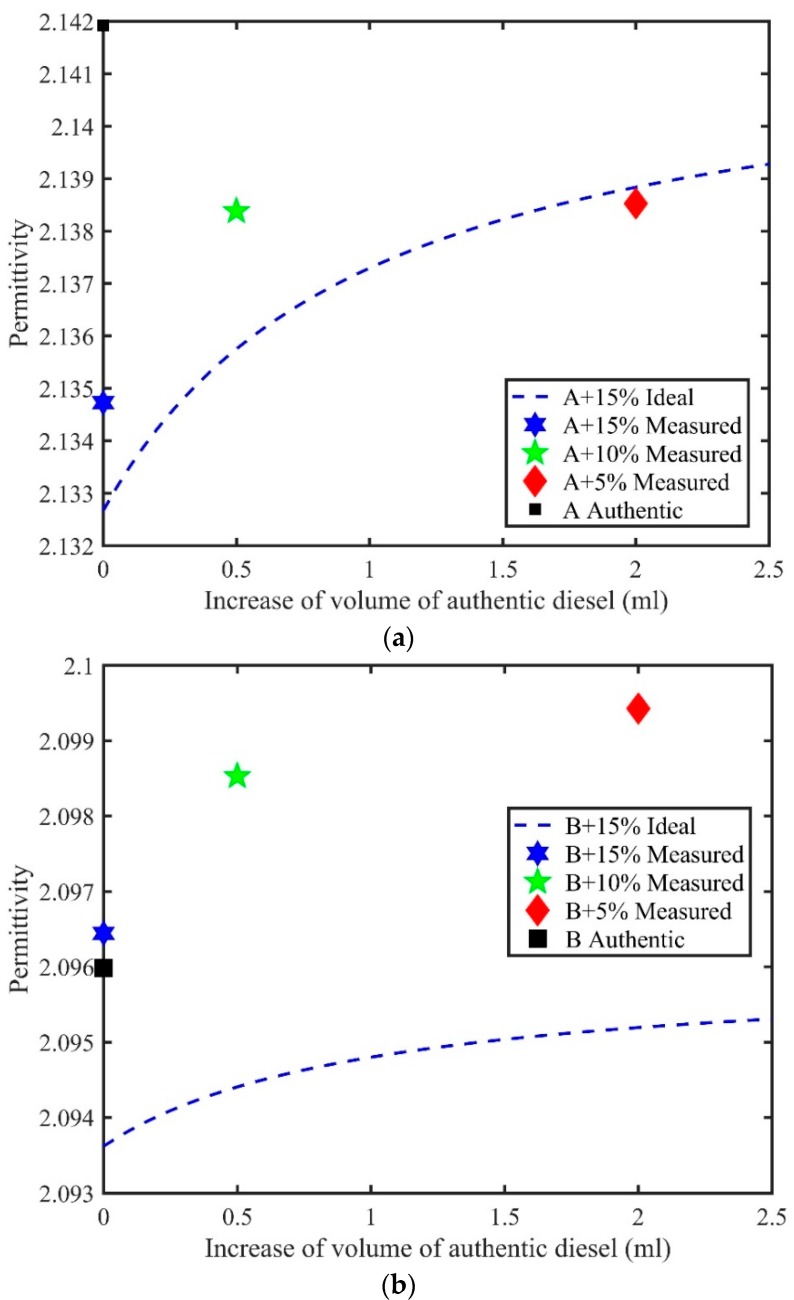
(**a**) Ideal permittivity for mixtures of sample A, and the measured permittivity values for adulterated samples obtained by the table model Abbe refractometer. (**b**) Ideal permittivity for mixtures of sample B, and the measured permittivity values for adulterated samples obtained by the table model Abbe refractometer.

We used the values given in Table 1 and Equation (3), to simulate the ideal permittivity (which is a reference) and compared that with the true permittivity of the adulterated samples. From Figure 1a, it can be observed that there were differences between the measured and ideal permittivities calculated from Equation (4) for sample A, hence showing excess permittivity. However, as one can expect when increasing the volume of authentic diesel, in this case to 2 V, the true permittivity value came closer and closer to the value of the ideal permittivity. Furthermore, the true permittivity of the authentic sample was clearly isolated from the true permittivity of the adulterated diesel oils. Moreover, the trend for increased volume showed that large volumes should be added if one is attempting to bring the value of adulterated sample closer to that of authentic fuel.

Next from Figure 1b, it can be observed that the situation for sample B was more striking although the refractive index values for sample B and kerosene were much closer to each other. Without prior knowledge, one could erroneously accept the 15% adulterated sample B as authentic diesel oil, if using only the close proximity of the refractive index readings of the two samples, as shown in Table 1. However, after mixing more diesel oil, the difference between the refractive index reading of the less adulterated and authentic sample became evident. Here, the difference between the measured and ideal permittivities was considerable, and even after increasing to 2 V, the difference between the ideal and true permittivities was big due to the different, stronger molecular interactions between sample B and kerosene than between sample A and kerosene. In the latter case, there was little excess permittivity. The excess permittivity of a binary liquid mixture can change sign (this is the case of Tanzanian samples) and magnitude depending on the volume fractions of the two liquids in the mixture, hence showing the complexity of the problem, which, in the present case, involves the screening of adulterated diesel oil.

### 4.2. Field Measurements in Tanzania

**Figure 2:**
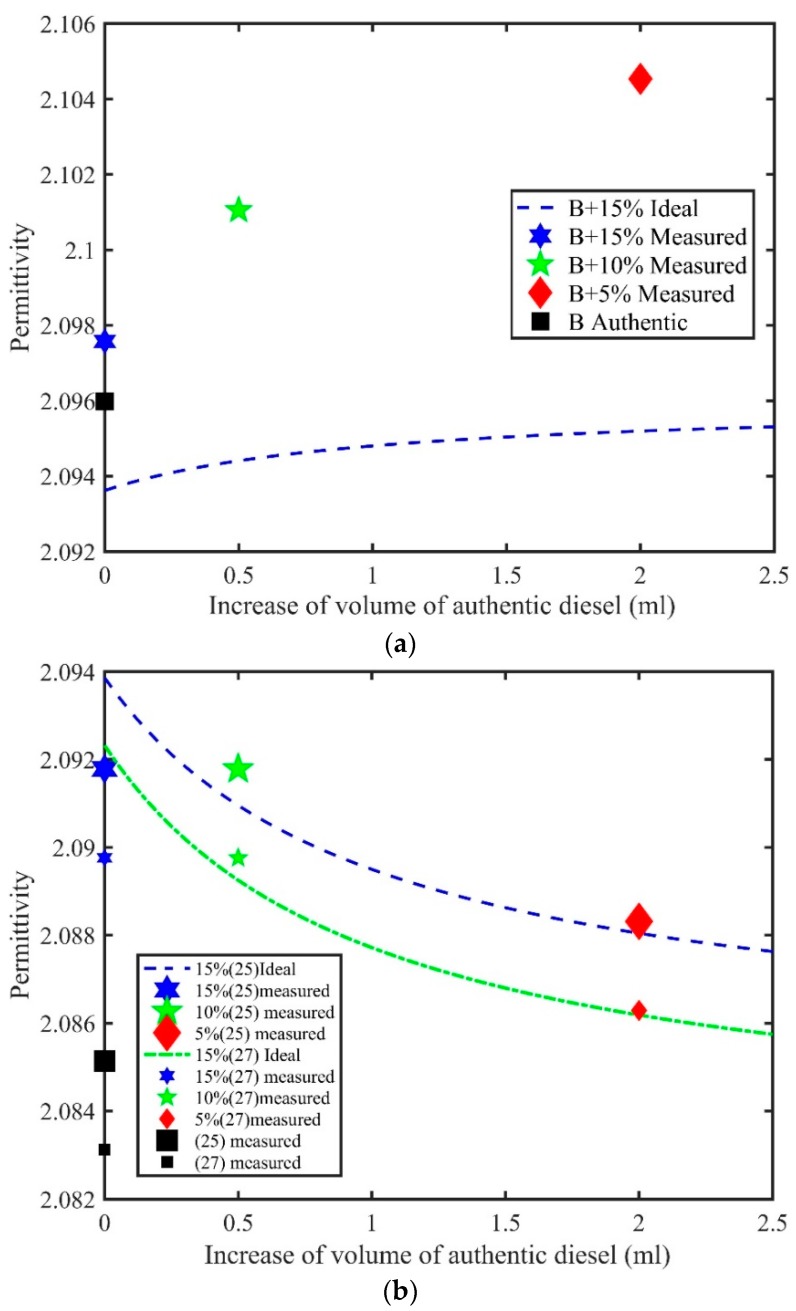
(**a**) Ideal permittivities for mixtures of sample B, and the true permittivity values for adulterated samples obtained by handheld Abbe refractometer data; (**b**) Ideal permittivity of authentic samples and the true permittivity of adulterated samples for Tanzanian fuels.

After using the training set and accurate table model of the Abbe refractometer for true permittivity studies as a function of volume increase, we studied the same training samples but using a less accurate hand-held refractometer to determine its feasibility for application in field measurements of adulterated diesel oil. Here, we show only data related to the most difficult case—the adulterated B sample (a similar data trend also holds for sample A). It is obvious from Figure 2a that the data for sample B in this figure were very similar to those shown in Figure 1b. This means that the hand-held device works consistently with the more accurate table model. Here, the difference between measured and ideal permittivity was also considerable, and even after increasing to 2 V, the difference between the ideal and true permittivities was rather big. Moreover, we observed that for this problematic sample, the hand-held model could be trusted for detecting adulteration and estimating its level by increasing the volume of authentic sample. From Figure 2a, it is possible to deduce that this method along with the hand-held refractometer could be applied even under circumstances where the refractive index of diesel is very close to that of kerosene, such as that of sample B. Hence, we obtained confidence to use the hand-held refractometer in field conditions and to exploit the measurement protocol of volume increase and related permittivity.

Next, we show an example of illustrating measurement data, which were obtained in field conditions in Tanzania with the aid of the hand-held refractometer. From Table 3, it can be observed that the refractive index values for the authentic diesel, 5% adulterated samples, and 10% adulterated sample all differed in the third decimal; also, the difference was in ascending order with respect to adulteration, namely authentic diesel oil had the lowest refractive index followed by 5%, and 10%. Furthermore, it can be observed that apparently, the hand-held refractometer was unable to differentiate between 10% adulteration and 15% adulteration as it gave the same value for both adulteration levels. This could result in the erroneous conclusion of an authentic diesel oil sample. However, when the volume of the adulterated sample was increased so that the adulteration level became 5%, the refractive index and naturally true permittivity were different from those in the two higher adulteration cases. Hence, we were able to conclude that the sample was a fake. This example clearly shows that the excess permittivity of two different adulterations can be the same, but after continuation of the dilution of the suspected sample by authentic diesel oil, the fake diesel oil will eventually be screened out. Note that here kerosene had a higher refractive index than diesel, whereas the opposite held for the Finnish samples. From Table 3, it is obvious that there was slight decrease in the refractive index readings for the samples as the temperature increased, while the obtained refractive index values for the adulterated samples lay in between those of authentic diesel and kerosene, in line with the assumptions of the conventional binary liquid mixing model. Nevertheless, at both measurement temperatures the message was clear—the refractive index readings for the 10% and 15% adulterated samples were practically the same.

Next, in Table 4, the estimated fill fractions of authentic diesel oil present in the mixture, calculated by using the Lorentz-Lorenz model from Equation (1), are shown, as well as the measured refractive index data from Table 3 at two different temperatures. For these samples, we obtained rather nice fill fraction estimates of the diesel oil at both temperatures for 5% adulteration; furthermore, the estimate for the 10% adulterated sample was almost perfect. However, the calculation gave us the same volume fraction estimate for both the 10% and 15% adulterated samples. This is natural because the hand-held refractometer gave the same refractive index reading for both the 10% and 15% adulterated samples. These refractive index values indicate that there was still strong intermolecular interaction present for the 10% adulterated sample but, this was becoming weaker for the 5% sample. The results for the fill fraction were as expected; in particular, the ideal Lorentz-Lorenz model works better for minimally adulterated than for highly adulterated samples.

Finally, Figure 2b shows the permittivity data from the Tanzanian samples at two different temperatures. It can be concluded again that there were intermolecular interactions between kerosene and diesel oil because of the departure of the true permittivity from the ideal permittivity, showing in this case both negative and positive excess permittivity. As already stated above, these samples are different from the Finnish samples in the sense that kerosene had a higher refractive index than diesel, whereas the opposite was true for the training sets. As a result, the curves for the ideal permittivity for Tanzanian samples shown in Figure 2b have a different trend from those shown in Figure 1 and Figure 2a. In the case of Figure 2, just as with sample A, the increase in diesel volume by 2 V gave a near match between the ideal and measured permittivities. The data points were rather similar at the two different temperatures. The 15% sample showed negative, the 10% sample showed positive, and the 5% sample showed nearly zero excess permittivity. Therefore, based on the observations made above, we propose that the hand-held refractometer, protocol of measurement and data analysis can be exploited in field conditions for the screening of fake diesel oils.

Diesel oil is a complex system containing hundreds of hydrocarbons [18]. Our goal was not to study the chemistry of the intermolecular interactions of diesel oil and kerosene molecules, but merely to monitor the excess permittivity of training sets, first in the laboratory, and thereafter, in samples in field measurement conditions. This study has clearly shown non-zero excess permittivity for relatively highly adulterated diesel oils, hence, resulting in the uncertainty of the validity of a traditional binary mixing law that can be used for assessing the kerosene volume fill fraction in adulterated diesel oil. The failure of assessing the volume fraction is most probable in cases involving highly adulterated diesel oil, but after introducing more and more authentic diesel oil in an adulterated sample, the classical mixing laws—not only Lorentz–Lorenz but also others (we have studies also Arago-Biot and Newton but do not present the results here)—start to work well. The most difficult case regarding the Lorentz–Lorenz model for assessing the adulterated diesel oil fill fraction occurs when both authentic diesel oil and kerosene have almost the same refractive index value, which was demonstrated in the case of Finnish diesel oil sample B. Yet, adulterated diesel oil can be screened even in such a problematic case by increasing the sample volume with authentic diesel oil and detecting the corresponding refractive index values. When the refractive index of such a sample is not between the values of authentic diesel oil and kerosene, it is a signal that the sample is adulterated diesel oil.

Finally, let us consider the example of a country like Tanzania where there are different importers of fuel. Under such circumstances, it is obvious that there will be authentic diesel oils of slightly varying properties based on the processing of crude oil as well as the oil field of origin [19]. We can collect all the different legitimate diesel oils and create a library of the ideal permittivity curves of diesel oil and kerosene mixtures with different adulteration percentages, for different measurement temperatures. Thus, any suspected sample can be identified based on the process demonstrated in this work.

## 5. Conclusions

In this paper, we presented a rather simple measurement and analysis method for the screening of diesel oils adulterated by kerosene. The idea was to use a simple and inexpensive refractometer to screen for fake diesel oils. By mixing suspected fake and authentic diesel oils, measuring the refractive index after each mixture and using the true permittivity measure, it was possible to identify adulterated diesel oil. This analysis took advantage of the availability of authentic diesel oil thanks to regulating authorities like EWURA. Furthermore, it future studies, even if the refractive index of kerosene is not known, the protocol described in this paper could be exploited by increasing the volume of the suspected sample with authentic diesel oil and detecting the resulting refractive index. If there is a change in the refractive index, it is most probably a sign of a fake oil. Using the true permittivity as a measure, which appears also in the Lorentz–Lorenz formula, the rate of change in the true permittivity as a function of volume increase can be used as a means for determining a fake. Naturally, if the kerosene is available, then the Lorentz–Lorenz model for the estimation of the fill fraction of diesel oil can be used, but it must be take into account that this model works better when the volume increase is high enough and when the refractive index difference between the authentic diesel oil and kerosene is high enough. A library of calibration curves similar to those shown in this paper could be created, including all the diesel oils supplied into a particular country, to assist in the process of screening for fake diesel oils.

Moreover, for developing and third world countries where sophisticated and state of the art fuel measurement equipment and laboratories is an issue, the cheap hand-held device used in this study coupled together with the method proposed in this work, could assist in resolving many fuel adulteration detection issues in the field. This would further reduce the number of samples required to be taken to the central measurement unit in a laboratory. Thus, only very problematic samples, for which there are no available calibration curves in the library, or which behave strangely, would need to be taken to the central measuring station for more thorough investigation. This could include optical measurements using table refractometer and spectrophotometric measurements. We therefore propose that the method described in this paper is applied for the field measurement of diesel oil purity.

## Figures and Tables

**Table 1:** Refractive index (n) data for authentic diesel oil samples A and B, and kerosene sample C, together with their mixtures.

Sample	Authentic Sample n	Volume Fraction of Kerosene	Adulterated Sample n
A	1.46353	5%	1.46237
		10%	1.46232
		15%	1.46107
B	1.44775	5%	1.44894
		10%	1.44863
		15%	1.44791
C	1.44230		

**Table 2:** Volume fill fractions occupied by authentic diesel oils in the adulterated samples, estimated using the Lorentz–Lorenz model for samples A and B.

Sample	Percentage of Adulteration	True Volume Fraction	Calculated Volume Fraction (Lorentz–Lorenz)
A	5%	0.95	0.946
	10%	0.90	0.943
	15%	0.85	0.885
B	5%	0.95	1.218
	10%	0.90	1.161
	15%	0.85	1.03

**Table 3:** Refractive index data for authentic diesel oil, kerosene and their mixtures, measured in Tanzania.

Sample	Adulteration Percent	Refractive Index n (25 °C)	Refractive Index n (27 °C)
Diesel	0%	1.4440	1.4433
	5%	1.4451	1.4444
	10%	1.4463	1.4456
	15%	1.4463	1.4456
Kerosene	0%	1.4640	1.4644

**Table 4:** Known volume fill fractions occupied by authentic Tanzanian diesel oils in the adulterated samples estimated using the Lorentz–Lorenz model.

Tanzanian Sample	Percentage of Adulteration	Known Volume of Diesel	Lorentz–Lorenz 25 °C	Lorentz–Lorenz 27 °C
0.95	5%	0.95	0.945	0.948
0.90	10%	0.90	0.884	0.89
0.85	15%	0.85	0.884	0.89
